# Network Analysis of Publicly Available RNA-seq Provides Insights into the Molecular Mechanisms of Plant Defense against Multiple Fungal Pathogens in *Arabidopsis thaliana*

**DOI:** 10.3390/genes14122223

**Published:** 2023-12-16

**Authors:** Cynthia Soto-Cardinault, Kevin L. Childs, Elsa Góngora-Castillo

**Affiliations:** 1 Unidad de Biotecnología, Centro de Investigación Científica de Yucatán, Mérida 97205, Mexico; cyntsc10@gmail.com; 2 Plant Biology Department, Michigan State University, East Lansing, MI 48824, USA; kchilds@msu.edu; 3 CONAHCYT-Unidad de Biotecnología, Centro de Investigación Científica de Yucatán, Mérida 97205, Mexico

**Keywords:** public RNA-seq datasets, weighted gene co-expression network analysis, gene module, arabidopsis, fungal pathogens, plant defense

## Abstract

Fungal pathogens can have devastating effects on global crop production, leading to annual economic losses ranging from 10% to 23%. In light of climate change-related challenges, researchers anticipate an increase in fungal infections as a result of shifting environmental conditions. However, plants have developed intricate molecular mechanisms for effective defense against fungal attacks. Understanding these mechanisms is essential to the development of new strategies for protecting crops from multiple fungi threats. Public omics databases provide valuable resources for research on plant–pathogen interactions; however, integrating data from different studies can be challenging due to experimental variation. In this study, we aimed to identify the core genes that defend against the pathogenic fungi *Colletotrichum higginsianum* and *Botrytis cinerea* in *Arabidopsis thaliana*. Using a custom framework to control batch effects and construct Gene Co-expression Networks in publicly available RNA-seq dataset from infected *A. thaliana* plants, we successfully identified a gene module that was responsive to both pathogens. We also performed gene annotation to reveal the roles of previously unknown protein-coding genes in plant defenses against fungal infections. This research demonstrates the potential of publicly available RNA-seq data for identifying the core genes involved in defending against multiple fungal pathogens.

## 1. Introduction

Pathogenic fungi are a significant factor in global crop production. Pathogen-related crop damage leads to annual losses ranging from 10% to 23%—equivalent to over USD 200 billion in economic damages [1,2,3]. Climate change has led to unprecedented challenges in food production, including the growing resistance to antifungal agents. In a warmer world, fungal infections are expected to become more widespread among crops, as the life cycle of fungi is directly influenced by temperature and humidity [4,5,6]. According to prediction models, the prevalence of pathogenic fungal infections in commercial crops will rise by 5% to 100% by 2050 as a result of climate change [7,8].

Pathogenic fungi can be classified into three groups: biotrophs, which feed on living tissues; necrotrophs, which kill the host cell and obtain nutrients from dead tissues; and hemibiotrophs, which exhibit an intermediate lifestyle, combining traits of both biotrophs and necrotrophs [9,10,11,12]. One highly important pathogen is *Colletotrichum higginsianum*, an ascomycete fungus with a hemibiotrophic nature. This fungus causes anthracnose, a disease that affects numerous monocotyledonous and dicotyledonous plants worldwide [9,13,14]. Another important pathogen, *Botrytis cinerea*, is a necrotrophic fungus with a broad range of hosts that has devastating effects on various plant tissues, including foliage, stems, flowers, and fruits [13,15,16,17].

Plants have developed complex molecular mechanisms to protect themselves from invading pathogens, including the ability to identify pathogens via pathogen-associated molecular patterns (PAMPs) with the help of pattern recognition receptors (PRRs). This leads to a process known as PAMP-triggered immunity (PTI), which limits the progression of disease. This pattern recognition is typically linked with calcium influx, callose deposition, bursts of reactive oxygen species (ROS), activation of miRNA pathways, activation of MAPK cascades, and increased expression of various defense-related genes, such as disease-related proteins (PR) [18,19,20]. In some cases, effectors produced by pathogens are recognized by plant resistance proteins, promoting a response known as effector-triggered immunity (ETI). One of the roles of ETI is to stimulate the hypersensitive response (HR), which results in rapid cell death at the site of pathogen invasion, limiting the spread of the pathogen. In addition, various signal transduction events, such as nitrous oxide, lipids, and various phytohormones that identify pathogen invasions, initiate plant immune defense responses [21,22,23,24]. The PTI and ETI immune responses are interconnected, as the activation of ETI improves the PTI signaling pathway. Both responses share functions related to the recognition of danger signals and the activation of defense mechanisms [18,25,26,27,28].

In the era of next-generation sequencing and machine learning, and considering the need for proactive responses to climate change, it is crucial to implement more comprehensive strategies in modern agriculture. One useful resource is the NCBI SRA, which contains more than 17 petabytes of public sequence data [29]. This underused data repository can be exploited using novel computational methods. A large portion of the sequence data is relevant to the agricultural sector, and opportunities are emerging to implement machine learning strategies that incorporate this immense pool of genomic data.

There is a growing interest in using public RNA-seq datasets to investigate intricate biological processes such as plant defense [30,31,32]. Publicly available RNA-seq expression data are a valuable resource for improving crop quality, particularly when conducting experiments under various environmental stressors or diseases is not feasible. However, the reutilization of public RNA-seq data is limited by the challenge of integrating batch datasets generated from different studies due to experimental variation, which can introduce bias; this must be carefully considered during analysis to ensure accurate and reliable results [30]. 

Traditionally, transcriptome studies with RNA-seq have been widely used for the identification of specific genes expressed during the fungal infection process, with a focus on specific characteristics of the plant or fungus [9,33,34,35,36,37]. In this study, our objective is to identify the core genes involved in defending against *B. cinerea* and *C. higginsianum* pathogens. We define core defense genes as those that confer a broad-spectrum defense mechanism and are not restricted to specific pathogens. We used publicly available RNA-seq expression information from the *C. higginsianum*–*A. thaliana* and *B. cinerea*–*A. thaliana* pathosystems. Both of these valuable models have been extensively studied with regard to specific responses during plant–pathogen interaction [14,15,16,17,38,39,40,41]. Additionally, only a limited number of studies have been conducted to understand common defense mechanisms against biotic stress, and our understanding of this topic is still at an early stage [42,43]. To date, the common reactions that these fungal pathogens can elicit in the host remain unclear.

One strategy, Weighted Gene Co-expression Network Analysis (WGCNA) [44], has contributed to crop improvement by revealing complex gene–gene interactions and helping to identify key genes that regulate important traits [45]. For instance, in soybeans, a gene module related to seed development was recognized and was found to include several genes involved in fatty acid biosynthesis [46]. In wheat, a set of genes associated with resistance to Fusarium head blight, a devastating fungal disease, was identified [47]. Furthermore, gene co-expression networks have been constructed using publicly available expression datasets. One of these studies used gene expression data to functionally annotate the rice proteome [48]. Moreover, other studies used gene co-expression networks to identify core genes in *A. thaliana* that respond to biotic stress using microarray data [42] and those in maize that respond to fungal infection using RNA-seq data [43]. However, neither of these studies considered batch-effect correction.

To identify core genes expressed during *C. higginsianum* and *B. cinerea* defense in *A. thaliana*, publicly available RNA-seq data were preprocessed for batch-effect correction using an in-house method, allowing us to obtain integrated gene expression data that could be used with the WGCNA [44]. Our preprocessing framework includes data integration, normalization and standardization based on variability identification while emphasizing a minimal loss of data, and it is based on statistics such as Gaussian distributions, kernel density estimation (KDE), and quantiles, which assist with the generation of evaluation metrics [49]. A module containing core genes that respond to infection with *B. cinerea* and *C. higginsianum* was identified. Using the DAVID agglomeration method [50], 33 genes were annotated by function across six categories, providing new insights into the functions of currently unknown protein-coding genes. Our findings demonstrate that publicly available RNA-seq data are a valuable resource for identifying broad-spectrum genes related to the defense against multiple fungal pathogens.

## 2. Materials and Methods

### 2.1. RNA-Seq Data Selection 

Twenty-five RNA-seq datasets of fungus-infected leaves of *A. thaliana* (Col-0) were obtained from the National Centre for Biotechnology Information’s Sequence Read Archive (NCBI SRA) database. The *C. higginsianum*–*A. thaliana* dataset was composed of 8 samples from BioProject accession number PRJNA148307 [9,51]; the *B. cinerea–A. thaliana* dataset comprised 6 samples from BioProjects with accession numbers PRJNA315516 and PRJNA593073; the *Sclerotinia sclerotiorum–A. thaliana* dataset consisted of 3 samples from BioProject accession number PRJNA418121 [52], and this dataset was used as an outlier control. The control dataset comprised 8 samples of healthy plants from BioProjects PRJNA315516 and PRJNA418121. The sample collection was performed from 12 to 30 h for control samples, and from 12 to 40 h for fungus-infected leaf samples (Supplementary Table S1). The transcriptomes were sequenced on Illumina platforms. The number of reads ranged from 10 to 30 million, with read lengths ranging from 93 to 150 bp (Table 1).

### 2.2. Sequence Quality Filtering and Expression Estimation

Reads from each dataset were processed for quality and filtered using FastQC v0.11.5 [53] and Trimmomatic v.0.38 [54] tools, using a Phred Score ≥20 and a minimum sequence length ≥40 bp. Filtered sequences were aligned to the *A. thaliana* genome TAIR10 [55] and the latest Araport 11 annotation (GenBank accessions CP002684–CP002688), using STAR alignment tool v2.7.5 [56], with parameters sjdbOverhang = 92 and genomeSAindexNbases = 7 to adjust the tool to the genome size and alignIntronMin = 8 and alignIntronMax = 1999 to adjust the minimum and maximum intron lengths [57]. The alignment quality reported in SAM/BAM files [58] were evaluated with the HTSeq-qa tool v0.11.1 and the expression abundance was estimated using the HTSeq-count tool v0.11.1 [59]. 

### 2.3. Data Preprocessing and Batch-Effect Correction

**Figure 1:**
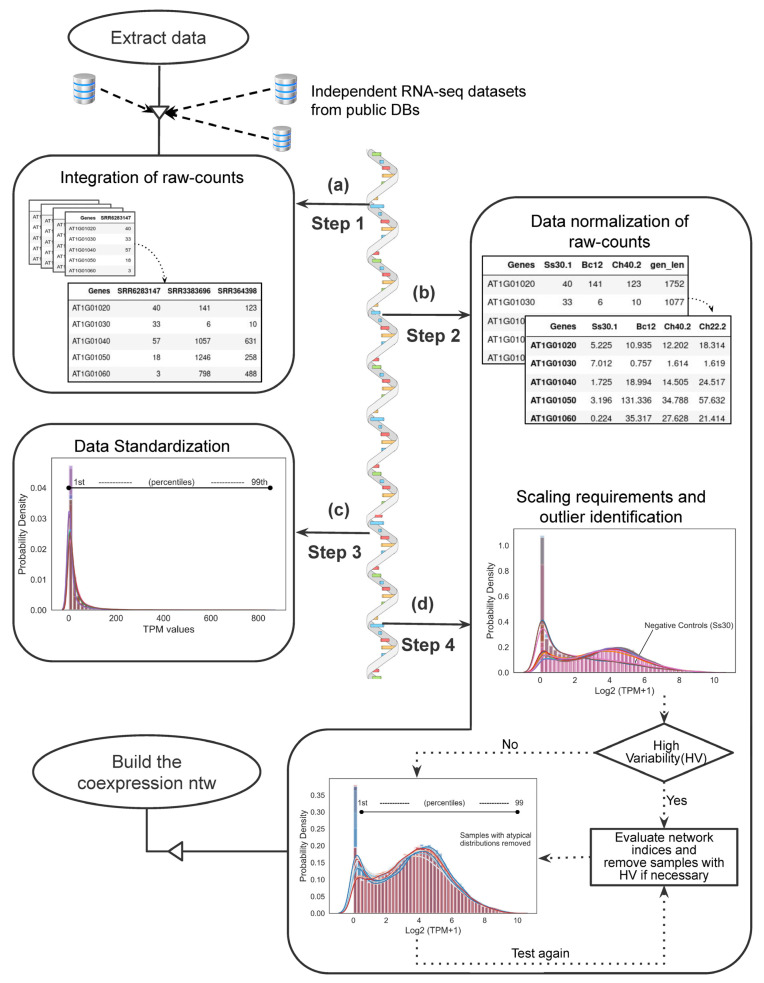
Framework for integrating publicly available RNA-seq data. (**a**) Step 1. Raw counts are integrated in a single expression matrix and evaluation metrics (standard deviation (σ), mean (µ) and range (R)) are calculated. Genes with zero expression values are removed. (**b**) Step 2. Data arrays are normalized by TPM and evaluation metrics are recalculated. (**c**) Step 3. Percentiles and distribution are calculated to identify tail values within the distribution. TPM values below the 1st and above the 99th percentile are removed, and evaluation metrics are recalculated. (**d**) Step 4. Normalized and filtered TPM values are transformed to log2(TMP + 1) to identify atypical distributions, and evaluation metrics are recalculated.

Central tendency and statistical dispersion measures were calculated, including the mean (µ), standard deviation (σ), and range (R), and histograms with KDE of distributions and percentiles were created using Seaborn [49] (Figure 1).

#### 2.3.1. Integration of Raw Counts and Data Normalization (Figure 1a,b)

A single expression matrix was generated using the raw count files for each dataset. Protein-coding genes (henceforth referred to as genes) with expression values equal to zero were eliminated. Transcripts per million (TPM) [60] were used to perform data normalization with the TPM normalization tool v0.9.1 using Gene_length_extraction_from_GTF.ipynb [61]. For TPM normalization, the gene length was obtained from the *A. thaliana* genome GTF/GFF file [55]. The script 1_Step1_integrating_raw_counts.ipynb and script 2_Step2_TPM_normalization.ipynb were used to organize the gene expression data and perform data normalization, respectively (Table S2). 

#### 2.3.2. Data Standardization and Outlier Identification (Figure 1c,d)

The gene expression values were divided into percentiles, and top and bottom extreme tails were calculated. Genes with TPM values between the 1st and 99th percentile were retained, while genes beyond this range were discarded. After filtering, TPM values were transformed to log_2_ (TPM + 1). To evaluate the efficiency of our methods in detecting samples with atypical distributions, we included 3 samples with known atypical distribution as negative controls. The 3_Step3_TPM_standardization.ipynb and 4_Step4_Log2_scale.ipynb scripts were used (Table S2). 

### 2.4. Weighted Gene Co-Expression Networks and Identification of Core Genes

Weighted gene co-expression network analysis (WGCNA v1.69-81) was performed using R package [44]. The *pickSoftThreshold* function according to the Scale Free Topology model fit was used to define the appropriate soft-thresholding power. The adjacency matrix was generated using a signed topological overlap matrix (TOM), along with dissimilarity values (1-TOM). Gene clusters were detected using a dynamic cutting algorithm with a minimum module size of 20 and a default cutoff height (0.99). Merged networks were derived at 0.1 distances. Pearson’s correlation analysis (r^2^ > 0.75) was used to identify modules linked to phenotype. The *moduleEigenes*, *cor*, corPvalueStudent functions were used to define module membership (MM) and gene significance (GS) with a cutoff of r^2^ > 0.75, r^2^ < 0.75 and *p*-value < 0.05.

To identify the core genes, genetic modules from healthy-control and infected-plant gene networks were compared. Modules in the infected-plant gene network with a difference >75% in genes (unique genes) were selected as exclusive gene modules for the infected-plant gene co-expression network. The correlation cutoff was set at R^2^ > 0.50. Gene modules that fit these criteria were selected as core genes.

### 2.5. Functional Annotation of the Gene Co-Expression Networks and Core Genes

A Gene Ontology (GO) overrepresentation test was performed using PANTHER v17.0 [62,63]. Gene modules with coefficients of R^2^ > 0.75 were selected, and a binomial test with Bonferroni correction was used to identify GO-Slim terms for biological process and molecular function GO modules [64,65] (http://geneontology.org/, accessed on 11 July 2023).

The core genes were analyzed using The Database for Annotation, Visualization and Integrated Discovery (DAVID, https://david.ncifcrf.gov/home.jsp, accessed on 15 July 2023). Nine of the fourteen DAVID annotation categories were used in this study. To identify closely related genes within the module, the DAVID Gene Functional Annotation tool applies a *kappa* score distribution and an agglomeration method based on heuristic fuzzy multiple-linkage partitioning. These methods are implemented through the tool’s web-based interface, and allow the user to select different levels of stridency, ranging from low to very high [66] (in this study, medium and high levels were selected). The selected gene groups were those repeated across the selection criteria. To identify the functional gene groups most relevant to the study (e.g., those of plants infected with pathogenic fungi), the DAVID tool uses the EASE score, which is a modified Fisher’s exact test, to assign an enrichment score to each annotated gene group. The cutoff was >0.80 [50,66]. 

The Arabidopsis Information Portal (Araport) through ThaleMine webtool (https://bar.utoronto.ca/thalemine/, accessed on 4 September 2023) was used to identify each *A. thaliana* gene [67,68].

### 2.6. Implementation

Data processing was implemented using Jupyter Notebooks [69] for Python v3.8.10 and WGCNA v1.69-81 [44] for R v4.1.0. The R scripts and code are freely available via GitHub at https://github.com/cyntsc/RNA-Seq-raw-integration (accessed on 28 September 2023) (DOI 10.5281/zenodo.7076416) (Table S2).

## 3. Results

To improve our understanding of the core genes involved in defending against multiple fungal infections, we conducted a comparative study using publicly accessible transcriptome data from *C. higginsianum*– and *B. cinerea*–*A. thaliana* pathosystems (Table 1 and Table S1). First, we controlled the batch effects of public data to reduce data variability using an in-house framework (Figure 1). Next, we performed a weighted gene co-expression network analysis (WGCNA) [44] of the preprocessed data to identify the core genes. These core genes were then subjected to further agglomerative analysis using DAVID [50] to identify functions in closely related gene groups.

### 3.1. Controlling the Batch Effect in Public RNA-seq Data to Construct Gene Co-Expression Networks (GCN)

**Figure 2:**
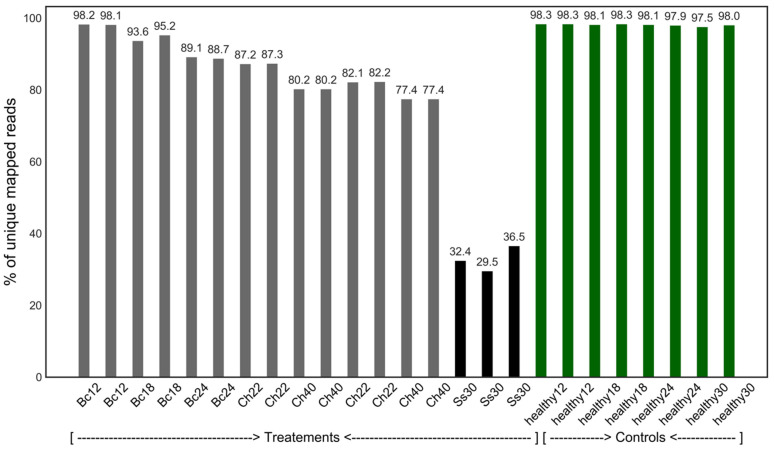
Rates of read alignments against the *Arabidopsis thaliana* genome. Percentage of unique reads mapped to the *A. thaliana* genome (TAIR11) from healthy-control and infected-plant samples.

RNA-seq sequences were filtered for quality, recovering more than 92% of the reads. These were aligned to the *A. thaliana* TAIR10 genome [55], achieving an alignment coverage rate of 97.5% to 98.3% for the healthy samples and 77.4% to 98.2% for the infected samples. *S. sclerotiorum* samples (Ss30, Ss30.1 and Ss30.2), with alignment coverages ranging from 29.5% to 36.5%, were included as an outlier control to track data changes (Table 1, Figure 2).

After read mapping, two gene expression matrices were obtained, one containing more than 221 thousand expression values (27,655 genes × 8 samples) derived from healthy-control samples and another containing more than 470 thousand expression values (27,655 genes × 17 samples) from infected-plant samples. Genes with an expression value equal to zero were eliminated (Figure S1), resulting in a total of 22,426 and 24,239 genes in the healthy-control and infected expression matrices, respectively.

**Figure 3:**
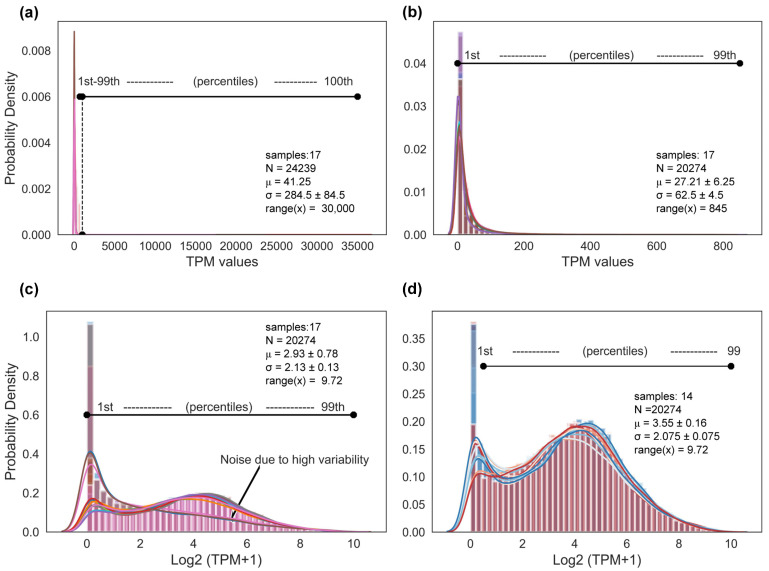
Integration of RNA-seq datasets. (**a**) The expression matrix was normalized to TPM and evaluation metrics were calculated, µ = 41.2, σ = 284.5 ± 84.5, and R *=* [0 to 30,000]. (**b**) Percentiles and distribution were calculated to identify tail values within the distribution. TPM values below the 1st percentile (TPM < 0.1) and above the 99th percentile (TPM > 845) were removed. Evaluation metrics were calculated (µ = 27.21 ± 6.25, σ = 62.5 ± 4.5 and R = [0 to 845]). (**c**) TPM values were transformed to log2(TMP + 1) to reduce the scale and to identify atypical distributions. Evaluation metrics were calculated (σ = 2.93 ± 0.78, µ = 2.13 ± 0.13, and R = [0 to 9.72]). (**d**) Fungal-infected samples with atypical distributions were removed (Ss30, Ss30.1 and Ss30.2) and evaluation metrics were calculated (σ = 2.075 ± 0.075, µ = 3.55 ± 0.16, and R = [0 to 9.72]). The colored lines represent the RNA-seq data used in this study; colored bars represent percentiles.

The expression values were normalized to TPM to set the means of the distributions to the same point, and the evaluation metrics showed a µ = 44.6, σ = 333 ± 6 and R = [0 to 36,543] for the healthy-control gene expression matrix and a µ = 41.2, σ = 284.5 ± 84.5 and R = [0 to 30,000] for the infected-plant gene expression matrix (Figure 3a). Extreme values in both expression matrices were identified and removed to reduce data variability. In the healthy-control expression matrix, 2262 genes were below the 1st percentile, in which TPM values were smaller than 0.1, and 372 genes were above the 99th percentile, with TPM values larger than 840. A total of 2634 genes were removed. In the infected-plant expression matrix, a total of 3965 genes were filtered. Of these, 3495 genes were below the 1st percentile and 470 genes were above the 99th percentile, where the TPM values were smaller than 0.1 and larger than 845, respectively. In total, 19,792 and 20,274 were obtained for the healthy-control and infected-plant expression matrices, respectively (Figure 3b). Data were transformed to log_2_(TPM + 1) to reduce the scale, and the metrics for the healthy-control samples were σ = 2.1 ± 0.1, µ = 3.4 ± 0.2 and R = [0 to 9.28] while for the infected-plant samples, the metrics were σ = 2.1 ± 0.1, µ = 2.9 ± 0.8 and R = [0 to 9.72] (Figure 3c).

To understand and demonstrate the effect of highly variable gene expression, Ss30, Ss30.1 and Ss30.2 samples were added to introduce variability in the data collection, with the aim of monitoring the efficacy of the workflow corrections. The histograms with KDE reveal a clear contrast between the preponderant and the outlier distributions of the Ss30, Ss30.1 and Ss30.2 samples (Figure 3c). The violin plots with KDE show individual distributions and reveal a pronounced difference in the distributions of the Ss30, Ss30.1 and Ss30.2 samples compared to other samples (Figure S2). Once the Ss30, Ss30.1 and Ss30.2 samples were eliminated, a more homogeneous dataset was obtained for the co-expression analysis (Figure 3d and Figure S2).

### 3.2. Gene Co-Expression Network Analyses and Gene Ontology Overrepresentation Test for Healthy and Infected Plants with Fungal Pathogens

**Figure 4:**
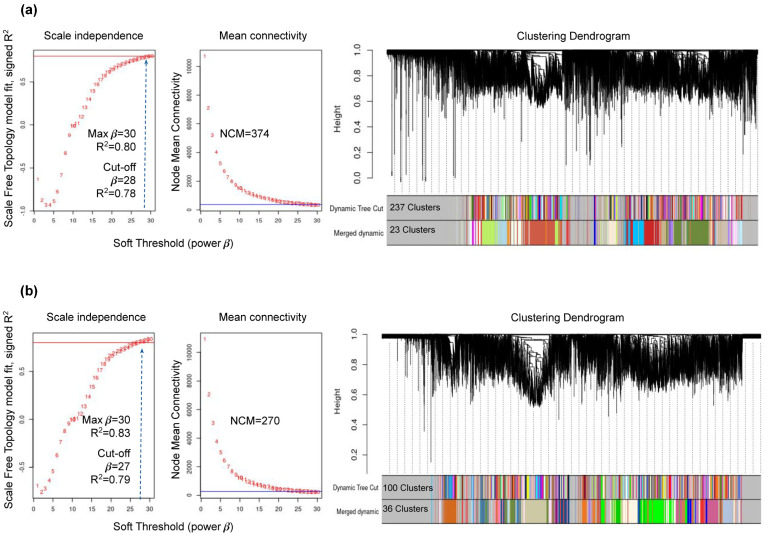
Co-expression network and clustering. The Scale-Free-Topology model was used to assess healthy-control samples and infected-plant gene networks. A signed network was built using Pearson’s method. (**a**) Control -sample gene network reached a maximum of R^2^ = 0.80. The soft threshold cutoff was set at β = 28 for network discovery, which resulted in R^2^ = 0.78 and a Node Mean Connectivity (NMC) of 374 genes. The merged network comprised 23 gene clusters. (**b**) Infected-plant gene network reached a maximum of R^2^ = 0.83. The soft threshold cutoff was β = 27, which resulted in a model fit of R^2^ = 0.79 with an NCM of 270 genes. The merged network contained 36 gene clusters.

Two gene co-expression networks were built. In the healthy-control gene network, a coefficient R^2^ = 0.80 was reached, and 237 clusters were identified with a Node-Connectivity Mean (NCM) of 374 genes per cluster (β = 28; R^2^ = 0.78). The merged network provided 23 clusters (Figure 4a, Table 2). In the infected-plant gene network, a coefficient R^2^ = 0.83 was reached, and 100 clusters with a NCM of 270 genes per cluster were identified (β = 27; R^2^ = 0.79). The merged network contained 36 clusters (Figure 4b, Table 2). A correlation of module eigengenes to disease trait data was performed, with a significant cutoff value of |GS| > 0.75 and *p*-value <0.05 (Datas S1 and S2).

To confirm the biological relevance of the identified networks, biological and molecular functions for each gene network were identified via a Gene Ontology (GO) overrepresentation gene test on the genetic modules with coefficients R^2^ > 0.75. Three and four genetic modules were analyzed to identify molecular function and biological processes from the healthy-control and infected-plant gene networks, respectively (Table S3). The number of genes in each module of the healthy-control sample gene network ranged from 79 to 2024, and 34–96% of these were assigned to ontology classes. In contrast, the infected-plant gene network modules contained 72 to 818 genes, 25–30% of which were classified into ontology classes (Table S4).

**Figure 5:**
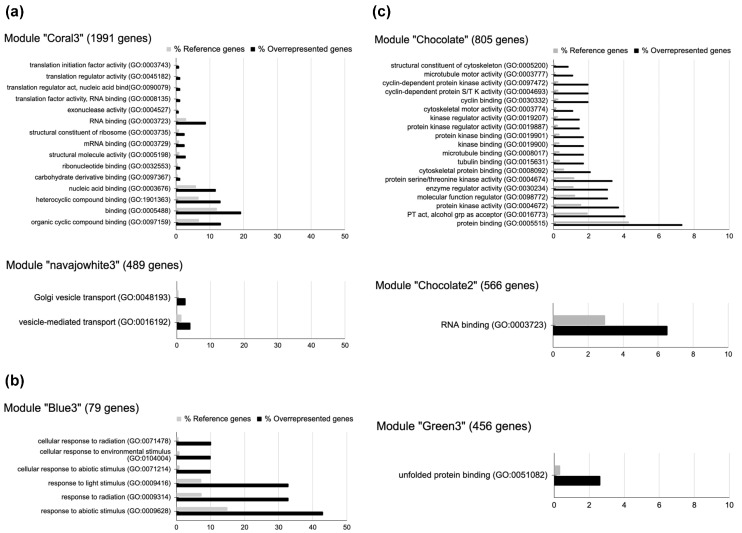
Gene Ontology (GO) overrepresentation test. (**a**) Bar plots of overrepresented molecular function GO-slim classes in Coral3 gene module and overrepresented biological process GO-slim classes in Navajowhite3 gene module obtained for control sample gene network. (**b**) Bar plot of overrepresented biological process GO-slim classes in Blue3 gene module obtained for control sample gene network. (**c**) Bar plots of overrepresented molecular function GO-slim classes in Chocolate, Chocolate2 and Green3 gene modules obtained for infected-plant gene expression network.

The results for the healthy-control gene network showed overrepresentation of 16 molecular functions mainly related to binding (GO:0005488) and translation (GO:0045182) in the “Coral3” module, which contains 1991 genes. The “Navajowhite3” module has 489 genes, and two biological processes related to vesicle-mediated transport (GO:0016192) and Golgi vesicle transport (GO:0048193) were overrepresented. The “Blue3” module contains 79 genes, and response to light (GO:0009416) and radiation (GO:0009314) are the main functions associated with this module (Figure 5a,b). In contrast the infected-plant gene network modules showed overrepresentation of 19 molecular functions in the 805-gene “Chocolate” module, in which protein binding (GO: 0005515) and kinase activity (GO: 0004672) are the main molecular functions. The “Chocolate2” and “Green3” modules each had only one molecular function class. The molecular function for the “Chocolate2” module is related to RNA binding (GO:0003723), while the “Green3” module is associated with unfolded protein binding (GO:0051082) (Figure 5c, Data S3).

### 3.3. Identification and Functional Annotation of Core Defense Genes in A. thaliana

**Figure 6:**
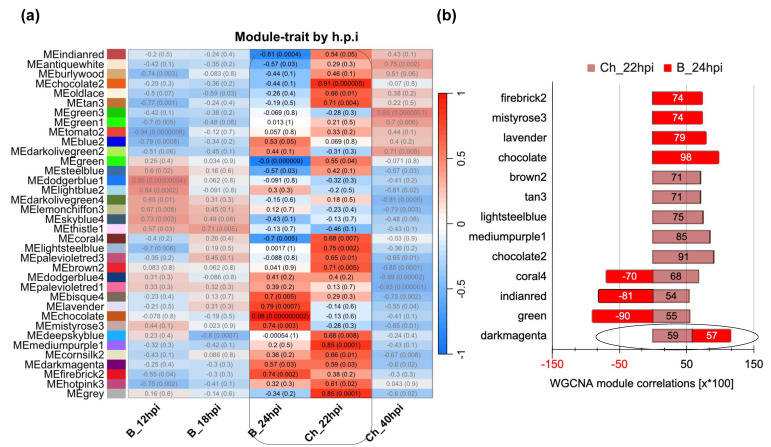
Core gene module. (**a**) The heatmap shows the different treatments analyzed in the study. The rectangle highlights the treatments with significant correlation. B_24 hpi represents the sample with *B. cinerea* at 24 h pos infection. Ch_22 hpi corresponds to the sample with *C. higginsianum* at 22 h postinfection. (**b**) Selected modules are displayed. The ellipses represent the consensus module “Darkmagenta”, with a R^2^ > 0.59 for *C. higginsianum* and a R^2^ > 0.57 for *B. cinerea*.

To identify defense core genes related to multi-fungal infections, healthy-control and infected-plant networks modules were compared, and modules in the infected-plant gene network with at least 75% unique or different genes were identified (Figure S3). Finally, modules that showed a positive correlation (R^2^ > 0.50) were selected. It was determined that the “Darkmagenta” module fit our criteria, with a correlation coefficient of R^2^ = 0.75 for *B. cinerea* at 24 hpi and a R^2^ = 0.59 for *C. higginsianum* at 22 hpi (Figure 6). It contains 113 genes, and of these, 103 (77.4%) were exclusively in this module (Figure S3). The functional annotation of this module was performed using the Database for Annotation, Visualization and Integrated Discovery (DAVID, https://david.ncifcrf.gov/home.jsp, accessed on 28 September 2023), which helps to identify closely related gene groups [50,66]. 

The 113 genes in the “Darkmagenta” module were mapped to nine DAVID functional categories. To categorize highly related genes into functional groups relevant to the pathogenic fungal infection, the DAVID gene functional annotation tool [66] employs a *kappa* distribution score, an agglomeration method and an EASE enrichment score. The web tool implementation enables users to select different stringency levels. We identified gene groups that remained consistent at medium and high stringency. Thus, 14 enriched functional groups were obtained and then filtered using an EASE score >0.80 and *p*-value < 0.05, resulting in six enriched functional groups (Table 3 and Table S5). Of these six groups, seven genes were classified in the “WD40/YVTN repeat-like-containing” group, three genes in the “sterol metabolism” group, ten genes in the “glycosyltransferase” group, seven genes in the “intracellular protein transport” group, four genes in “Zinc finger, FYVE/PHD-type”, and six genes in “Methyltransferase” (Table 3). Four of these thirty-seven genes have multiple functions (AT5G06050, AT5G13960, AT5G40870 and AAT5G45660) and are associated with at least two functional groups (Table 4). Finally, 33 defense-related genes and their roles in *B. cinerea* and *C. higginsianum* infection in *A. thaliana* at 22 and 24 hpi were identified through DAVID functional annotation (Table 4).

## 4. Discussion

Pathogenic fungi-induced diseases are among the main causes of losses in commercial crops. In light of major challenges such as climate change and fungicide resistance, it is imperative to develop global strategies to tackle these losses. Conducting experiments under a wide range of environmental stresses can be both challenging and time consuming. However, the advent of NGS technologies has allowed the collection of large amounts of data that could help to address the challenges associated with modern agriculture. The utilization of publicly available RNA-seq expression data holds great potential for further study of complex plant–pathogen interactions, leading to the identification of candidate genes and molecular markers and optimization of breeding strategies by targeting specific genes. However, utilizing publicly available RNA-seq data remains challenging. To tackle this, we based our study on computational approaches using public data from RNA-seq transcriptome studies of *A. thalian-B. cinerea* and *A. thaliana*-*C. higginsianum* pathosystems to identify core genes that respond to multiple types of fungal infection using GCN. Reanalysis of public RNA-seq expression data requires the minimization of technical biases, known as the batch effect, to obtain high-quality co-expression networks [30]. Although several tools may be used for batch-effect correction (such as ComBat-Seq [70], ComBat [71], svaseq [72]), further efforts may be required, particularly when sample sizes are limited (<30) [30]. Therefore, we developed a straightforward workflow to preprocess data in order to correct the batch effect that allowed us to recover over 80% of genes for WGCNA (Figure 1).

Previous studies revealed that gene modules generated by WGCNA from condition-dependent gene expression experiments are more informative than gene modules identified by combining the entire dataset regardless of condition [48]. Thus, two gene co-expression networks were constructed using filtered data from healthy (control) and infected *A. thaliana* plants with *B. cinerea* and *C. higginsianum*. The network for healthy-control plants included 23 gene modules with an NCM of 374, while for the network for infected plants, 36 gene modules with an NCM of 270 were obtained (Figure 4, Table 2). Additionally, the quality of each network was confirmed through GO overrepresentation analysis, resulting in biological processes and functions that are overrepresented in each network and linked to each phenotype. Modules with an R^2^ values greater than 0.75 were selected for both networks, and it was determined that within the healthy-control plant network, genetic modules coral3, blue3 and navajowhite3, modules with positive correlation (R^2^ of 0.98, 0.96, and 0.79, respectively) were related to plant development and growth, exhibiting functions such as translation and protein binding (GO:0005488), vesicle-mediated protein transport (GO:0016192), as well as response to light and radioactivity (GO:0016192) (Figure 5, Table S2). The gene modules from the plant-infected network, chocolate, dodgerblue1, green and chocolate2 with positive correlations (R^2^ of 0.98, 0.96, 0.95, 0.91, respectively), displayed overrepresented molecular functions of genes associated with regulating gene expression and signal transduction, which are the primary stress response mechanisms. These functions included protein binding (GO:0005515), kinase activity (GO:0004672), RNA binding (GO:000373) and unfolded protein binding (GO:0051082) (Figure 5, Table S2) [73,74].

To identify core genes that respond to *B. cinerea* and *C. higginsianum* infection in *A. thaliana*, the genetic modules in the network of infected plants should differ by at least 75% compared to the network of healthy-control plants with a positive correlation (R^2^ > 0.5) for the infection conditions. Therefore, we selected the “Darkmagenta” module due to its differentiation in 77.4% of genes compared to the control. This module displayed an R^2^ = 0.57 with the *B. cinerea* treatment at 24 hpi and an R^2^ = 0.59 with the *C. higginsianum* treatment at 22 hpi, as shown in the heatmap (Figure 6). This module contained 113 genes that were functionally annotated using the DAVID tool [66], revealing 33 genes distributed across six functional categories (Table 3, Table 4 and Table S5).

The first group of genes in the “Darkmagenta” module consisted of seven genes (AT5G23430, AT3G13340, AT3G18060, AT5G51980, AT1G79990, VPS and VSC) that contained the WD40/YVTN repeat-like domain (IPR015943). In *A. thaliana*, 237 proteins have been reported to contain four or more copies of the WD40 domain and participate in diverse biological processes, including defense against pathogens [75,76]. The most extensively studied of the WDR proteins are the Gβ proteins, which are associated with type-1 membrane receptors in the plant innate immunity signaling pathway. Interactions between Gβ and type-1 membrane receptors convert extracellular signals into intracellular chemical defense responses, including reactive oxygen species (ROS) production, callose deposition, MAPK activation, defense gene activation and programmed cell death [77]. In this group, we identified at least 3 Gβ-type genes (AT5G23430, AT5G51980, AT1G79990) (Table 4). 

In the second group, we identified three CPG for key enzymes in sterol biosynthesis, SMT1, SMO1-1, and 3BETAHSD/D1 (Table 4). Sterols are a complex mixture of organic compounds that are synthesized in plants and function as structural components of cell membranes. They play a vital role in processes such as channel regulation, protein trafficking, signal transduction and plant–pathogen interactions [78]. The SMT1, 4-methyl sterol oxidase (SMO) and 3-BETAHSD enzymes participate in the biosynthesis of c24-alkyl sterol and cholesterol in plants [79]. The role of sterols in plant defense has been studied more comprehensively in relation to abiotic stressors, such as drought, salinity, and cold, than against biotic stress. However, certain sterols such as cholesterol activate biotic stress response genes [78], while pathogenic bacteria and ROS stimulate biosynthesis of stigmasterol. Moreover, PATHOGENESIS-RELATED PROTEIN 1 (PR-1) can bind various sterols, including stigmasterol, to inhibit pathogen growth by sequestering sterols from pathogens [80]. Although there is evidence suggesting that sterols might function as a crucial element in plant defense, further studies are needed to demonstrate their efficacy.

The glycosyltransferase genes in group three consist of 10 CPG (ALG3, FUT13, CALS1, UK/UPRT1, AT4G38040, GUT2, PARVUS, SETH2, AT5G45660, AT1G34270). Glycosylation is one of the major posttranslational modifications in plants, playing an important role in defense against pathogens by inactivating toxic microbial compounds and strengthening the cell wall. Glycosylation also regulates secondary metabolite levels by increasing their water solubility to facilitate metabolite transport throughout the plant, increasing the metabolites’ stability to avoid degradation and even altering their biological activity to increase toxicity to pathogens or decrease bioactivity for plants. An example of this is the glycosylation of salicylic acid (SA), a phytohormone that functions as a signal molecule during plant defense against pathogens, activating systemic acquired resistance (SAR). This glycosylation is believed to facilitate detoxification of SA, allowing it to be safely stored and moved within the plant, which helps to avoid excessive activation of defense responses that could harm the plant’s growth and development [81,82,83]. Interestingly, within this group of genes, we have identified CALS1 (AT1G05570), which responds to SA (Table 4).

Four genes were identified as Zinc FYVE/PHD group (EMB1135, ATX2, AT5G12350, and AT1G5062). The zinc finger protein (ZFP) transcription factors (TFs) constitute a vast family in the plant kingdom and play a crucial role in stress tolerance. The ZFP TFs mainly regulate genes involved in antioxidation activity, which reduce ROS accumulation. Controlling the ROS level is critical, as it helps to avoid damage in molecules, including DNA, proteins and lipids, and to enhance stress tolerance. AT1G50620 and EMB1135 (alternatively known as the FGT 1 gene, AT1G79350), containing the RING/FYVE/PHD-type (RFP) domain (IPR011011) [84,85], have a zinc ion binding function, and recent studies indicate that this function may help with ROS scavenging [85,86]. Additionally, rapid accumulation of ROS during the development of fungal appressoria has been observed as a defense mechanism to prevent penetration of the pathogenic fungi [87,88]. This is consistent with the infection period of *C. higginsianum,* since at 22 h post inoculation, the fungus is in the prepenetration stage and is engaged in forming the appressorium [9]. Additionally, FGT 1 (EMB1135) encodes the FORGETTER 1 protein that binds directly to a specific class of heat-inducible genes through nucleosome remodeling [89]. These types of ZFP TFs regulate cellular responsiveness by binding to promoter regions of actively expressed genes in a heat-dependent fashion, such as heat shock proteins (HSP). The HSPs are induced by a variety of stresses, including oxidative stress; however, the crosstalk between abiotic and biotic stress responses in the ROS network remains poorly understood [90].

A group of six methyltransferases were identified (AT3G15530, SUVH4, AT2G34300, SMT1, ATX2, AT5G06050); all of these genes are involved in methylation. However, to date, there have been few studies on the role of methylation in plant–pathogen interactions, particularly with regard to infections caused by pathogenic fungi. The available evidence suggests that epigenetic control of gene expression contributes to the fine-tuning of plant defenses in response to pathogens [91,92]. Crespo-Salvador et al. (2018) demonstrated that differentially expressed genes responding to *B. cinerea* infection were induced 24 h after infection and displayed similar histone modification patterns, suggesting that epigenetics marks may impact the transcriptional regulation in this particular pathosystem [93]. *A. thaliana* hypomethylated mutants showed resistance against the biotrophic pathogen *Hyaloperonospora arabidopsidis*, whereas hypermethylated mutants were more susceptible to this pathogen [92,94]. Additionally, research on geminivirus infections has shown that the SUVH4 protein is a virus-silencing target that aims to evade host defenses [95]. Interestingly, our results highlighted the presence of SUVH4 (AT5G13960) and ATX2 (AT1G05830), which are involved in the maintenance of epigenetic control [96].

## 5. Conclusions

Core gene clusters that respond to multiple fungal infections were identified. However, further studies are needed to elucidate the role of several important genes in plant immune responses. Identifying coexpressed gene associations may provide insights into the functions of currently unknown coding genes. In the context of climate change, genomic data repositories are important resources for developing new tools for crop improvement and disease prevention, positively influencing productivity, sustainability and food security.

## Figures and Tables

**Table 1:** Description of public RNA-Seq datasets downloaded from the SRA.

BioProject	Sample ID	HPI ^1^	Run	Layout	Reads (M ^2^)	% Clean Reads	% Aligned Reads
PRJNA148307	Ch22	22	SRR364389	SE	12.6	92.12	87.17
	Ch22.1		SRR364390	SE	12.4	92.03	77.45
	Ch22.2		SRR364391	SE	12.4	92.18	77.4
	Ch22.3		SRR364392	SE	12.2	92.08	87.29
	Ch40	40	SRR364400	SE	11.9	91.40	82.08
	Ch40.1		SRR364401	SE	11.9	91.40	82.25
	Ch40.2		SRR364398	SE	13.2	92.55	80.25
	Ch40.3		SRR364399	SE	13.2	92.73	80.25
PRJNA315516 PRJNA593073	Bc12	12	SRR3383696	SE	12.1	100.00	98.2
	Bc12.1		SRR3383697	SE	15	100.00	98.14
	Bc18	18	SRR3383779	SE	10.3	97.37	93.55
	Bc18.1		SRR3383780	SE	13.6	97.26	95.2
	Bc24	24	SRR10586397	PE	22.2	95.53	89.07
	Bc24.1		SRR10586399	PE	22	95.70	88.69
PRJNA418121	Ss30	30	SRR6283146	SE	20.8	95.01	36.48
	Ss30.1		SRR6283147	SE	20.9	96.09	32.35
	Ss30.2		SRR6283148	SE	21.2	92.90	29.5
PRJNA315516 PRJNA418121	healthy12	12	SRR3383640	SE	10.9	97.12	98.31
	healthy12.1		SRR3383641	SE	22.6	97.50	98.34
	healthy18	18	SRR3383782	SE	29.8	97.62	98.13
	healthy18.1		SRR3383783	SE	14.2	97.69	98.34
	healthy24	24	SRR3383821	SE	15	97.18	98.09
	healthy24.1		SRR3383822	SE	10.2	95.98	97.87
	healthy30	30	SRR6283144	SE	22.1	95.31	97.51
	healthy30.1		SRR6283145	SE	19.7	95.72	97.99

^1^ Hours Post Inoculation; ^2^ Million reads.

**Table 2:** TOM network results and the Node-Connectivity Mean (NCM).

Condition	R^2^	# Clusters of Standard Network	NCM	# Clusters of Merged Network
Healthy-control samples	β = 28; 0.78	237	374	23
Infected-plant samples	β = 27; 0.79	100	270	36

**Table 3:** Enriched functional groups from “Darkmagenta” gene module.

Category	Term (Group #)	*p* Value	P Benjamin	#Genes	Gene	E. Score
IPR015943	WD40/YVTN repeat-like-containing (group 1)	0.0029	0.5307	7	AT5G23430, AT3G13340, AT3G18060, AT5G51980, VPS11, AT1G79990, VCS	1.5527
KW-1207 KW-0752 GO:0016126	Sterol metabolism (group 2)	0.0125 0.0275 0.0277	0.4613 0.7459 0.9995	3	SMT1, SMO1-1, 3BETAHSD/D1	1.3511
GO:0016757 KW-0328	Glycosyltransferase (group 3)	0.0113 0.0346	0.8358 0.7467	10	ALG3, FUT13, CALS1, UK/UPRT1, AT4G38040, GUT2, PARVUS, SETH2, AT5G45660, AT1G34270	1.3489
KW-0968 GO:0006886	Intracellular protein transport (group 4)	0.0066 0.0428	0.1592 0.9999	7	PLA2-α, PAT2, AT1G60070, AT4G13730, AT1G14910, VPS11, AT1G79990	1.1112
IPR011011 IPR019787	Zinc finger, FYVE/PHD-type (group 5)	0.0190 0.0477	0.9928 0.9999	4	EMB1135, ATX2, AT5G12350, AT1G50620	1.0173
KW-0489	Methyltransferase (group 6)	0.0403	0.7991	6	AT3G15530, SUVH4, AT2G34300, SMT1, ATX2, AT5G06050	0.8795

**Table 4:** Description of core genes involved in multiple fungal infections.

Group	TAIR ID	Gene	Chr	Description
1	AT5G23430	AT5G23430	Chr5	Transducin/WD40 repeat-like superfamily protein.
1	AT3G13340	AT3G13340	Chr3	Transducin/WD40 repeat-like superfamily protein
1	AT3G18060	AT3G18060	Chr3	Transducin/WD-40 repeat family protein
1	AT5G51980	AT5G51980	Chr5	Transducin/WD40 repeat-like superfamily protein
1–4	AT1G79990	AT1G79990	Chr1	Coatomer subunit β-2. WD repeat COPB2 family
1–4	AT2G05170	VPS11	Chr2	Vacuolar protein sorting 11
1	AT3G13300	VCS	Chr3	VARICOSE. Transducin/WD40 repeat-like superfamily protein
2–6	AT5G13710	SMT1	Chr5	Sterol methyltransferase 1
2	AT4G12110	SMO1-1	Chr4	Sterol-4alpha-methyl oxidase 1-1
2	AT1G47290	3BETAHSD/D1	Chr1	3beta-hydroxysteroid-dehydrogenase/decarboxylase isoform 1
3	AT2G47760	ALG3	Chr2	Asparagine-linked glycosylation 3
3	AT1G71990	FUT13	Chr1	Fucosyltransferase 13
3	AT1G05570	CALS1	Chr1	Callose synthase 1
3	AT5G40870	UK/UPRT1	Chr5	Uridine kinase/uracil phosphoribosyltransferase 1
3	AT1G27440	GUT2	Chr1	Exostosin family protein
3	AT5G12350	AT5G12350	Chr5	Exostosin family protein(AT4G38040)
3	AT1G05570	CALS1	Chr1	Uridine kinase/uracil phosphoribosyltransferase 1(UK/UPRT1)
3	AT1G19300	PARVUS	Chr1	Nucleotide-diphospho-sugar transferases superfamily
3	AT3G45100	SETH2	Chr3	UDP-Glycosyltransferase superfamily protein
3	AT4G38040	AT4G38040	Chr4	Exotosin family protein. Glycosyltransferase 47 family
3	AT5G45660	AT5G45660	Chr5	Adenine phophoribosyltransferase
3	AT1G34270	AT1G34270	Chr1	Exotosin family protein
4	AT2G06925	PLA2-α	Chr2	Phospholipase A2 family protein
4	AT3G55480	PAT2	Chr3	Protein affected trafficking 2
4	AT1G60070	AT1G60070	Chr1	Adaptor protein complex AP-1
4	AT4G13730	AT4G13730	Chr4	Ypt/Rab-GAP domain of gyp1p superfamily protein
4	AT1G14910	AT1G14910	Chr1	ENTH/ANTH/VHS superfamily protein
5	AT1G79350	EMB1135	Chr1	RING/FYVE/PHD zinc finger superfamily protein
5–6	AT1G05830	ATX2	Chr1	Trithorax-like protein 2
5	AT5G12350	AT5G12350	Chr5	RCC1 family with FYVE zinc finger domain-containing protein
5	AT1G50620	AT1G50620	Chr1	RING/FYVE/PHD zinc finger superfamily protein
6	AT5G13960	SUVH4	Chr5	Histone-lysine N-methyltransferase, H3 lysine-9
6	AT3G15530	AT3G15530	Chr3	S-adenosyl-L-methionine-dependent methyltransferases superfamily
6	AT2G34300	AT2G34300	Chr2	S-adenosyl-L-methionine-dependent methyltransferases superfamily
6	AT5G06050	AT5G06050	Chr5	Putative methyltransferase family protein

## Data Availability

The code developed for this study is freely available via GitHub at https://github.com/cyntsc/RNA-Seq-raw-integration (accessed on 28 September 2023) (DOI 10.5281/zenodo.7076416).

## Supplementary Materials

The following supporting information can be downloaded at https://www.mdpi.com/article/10.3390/genes14122223/s1: Figure S1: Venn diagrams for nonexpressed genes; Figure S2: Distribution of atypical samples in infected-plant gene networks; Figure S3: Unique genes identified in infected-plant gene modules; Table S1: Sample information obtained from NCBI SRA; Table S2: Databases, Bioinformatic tools and source code; Table S3: Significant gene modules identified in healthy-control and fungal-infected samples; Table S4: Gene Ontology overrepresentation test summary; Table S5: Genes functional groups related to multiple fungal infections identified by DAVID; Data S1: Normalized expression data for control samples; Data S2: Normalized expression for infected-plant samples; and Data S3: Gene Ontology overrepresentation analysis results.
