# Supplementary material for: Network Analysis of Publicly Available RNA-seq Provides Insights into the Molecular Mechanisms of Plant Defense against Multiple Fungal Pathogens in Arabidopsis thaliana
Source: Genes (Basel). 2023 Dec 16;14(12):2223. doi: 10.3390/genes14122223 (PMC10743233; doi:10.3390/genes14122223)
Supplement: Supplementary file 1 [file genes-14-02223-s001.zip › Supplemental_material_CSC_Genes2023.pdf]

Network analysis of publicly available RNA-seq data provides insights into the molecular mechanisms of plant defense against multiple fungal pathogens in *Arabidopsis thaliana*

Cynthia Soto-Cardinault[1], Kevin L. Childs[2], Elsa Góngora-Castillo[3,*]

[1] Unidad de Biotecnología. Centro de Investigación Científica de Yucatán. Mérida, Yucatán 97205. México; cyntsc10@gmail.com

[2] Plant Biology Department. Michigan State University, East Lansing, MI 48824. USA; kchilds@msu.edu

[3] CONAHCYT-Unidad de Biotecnología. Centro de Investigación Científica de Yucatán. Mérida, Yucatán 97205. México

*Correspondence: elsa.gongora@cicy.mx

Supplemental Material

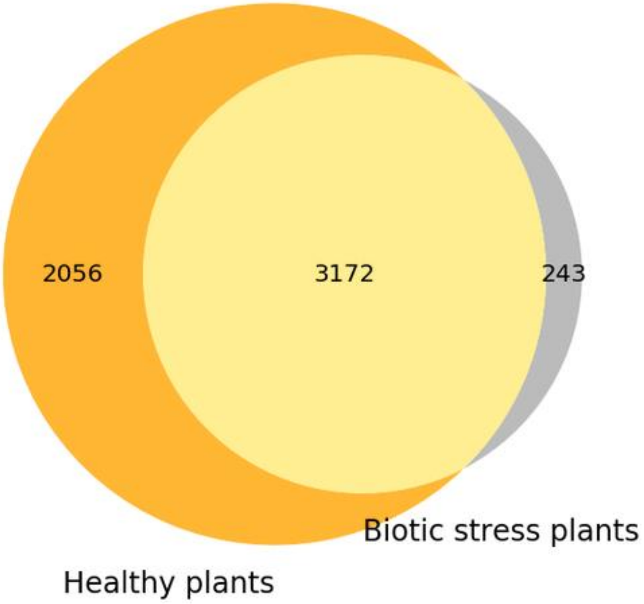

Figure S1. Venn diagrams for non-expressed genes. Expression values equal to zero were removed from both control sample and stress-treated sample datasets.

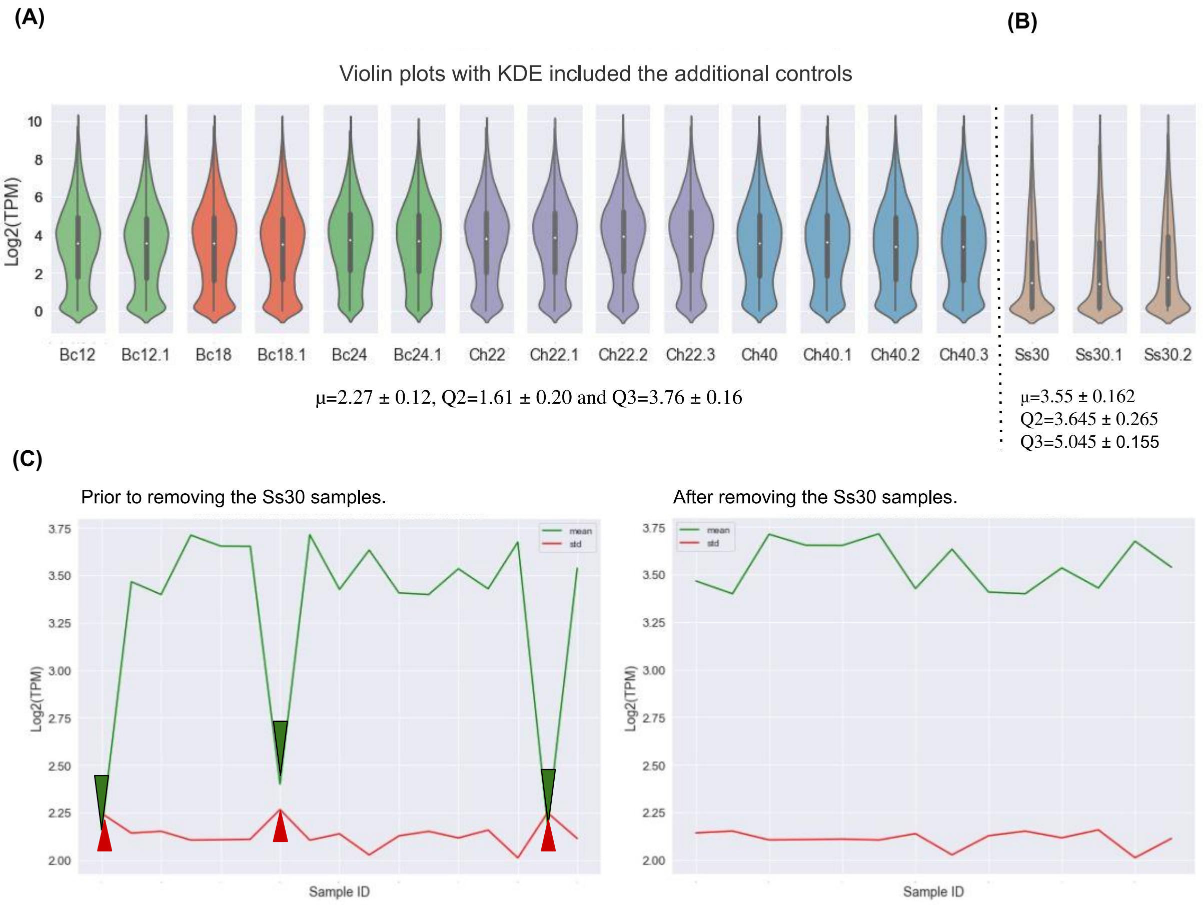

Figure S2. Distributions of atypical samples in stress-treated gene networks. (A) Distribution of stress-treated samples without including the Ss30s samples, **(B)** Distribution of the atypical samples, **(C)** Mean (green line) and the standard deviation (red line) before and after removing the Ss30, Ss30.1 and Ss30.2 samples from the stress-treated dataset.

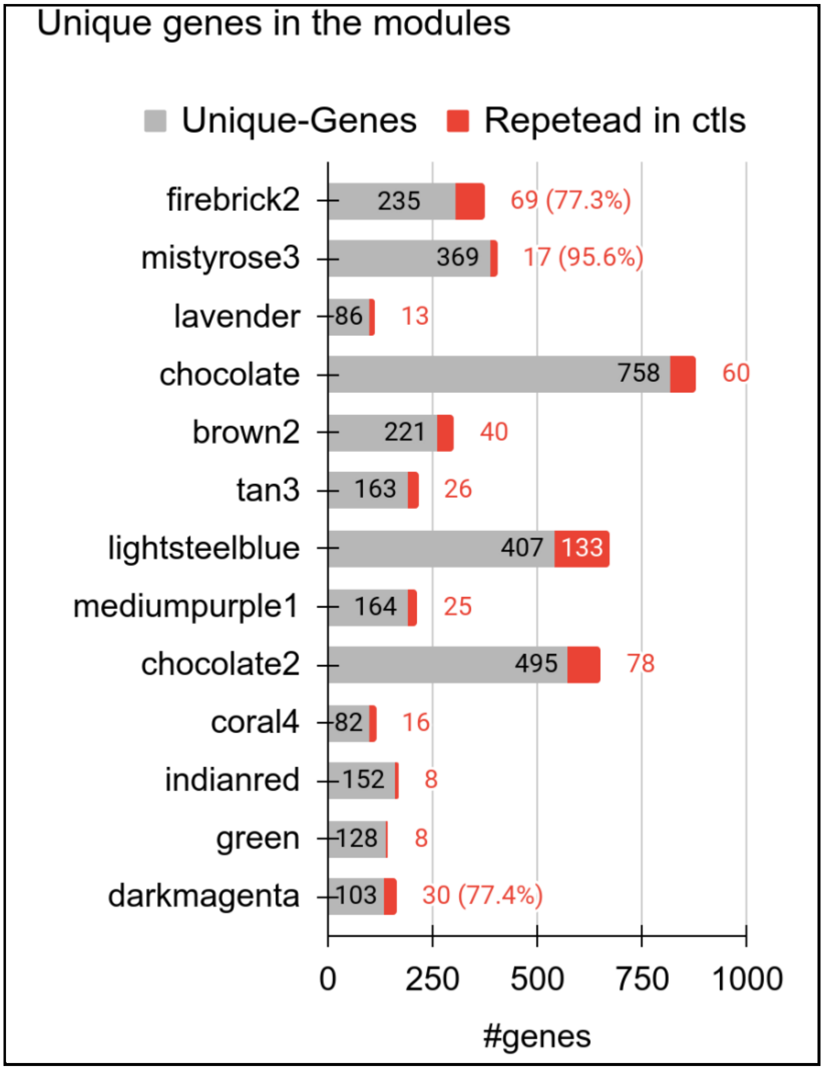

Figure S3. **Unique genes identified in infected-plants gene modules**. The gray bars show the number of genes that were unique in each gene module. The red bar show the number of repeated genes found in the healthy-control network when comparing both gene co-expression networks.

Table S1. Sample information obtained from NCBI SRA.

Sample	Treatment	HPI	Sample ID	BioProject	Reference
Arabidopsis thaliana (Col-0) leaves	*Collechotricum higginsianum*	22	Ch22	PRJNA148307	[1,2]
		40	Ch40		
	Sclerotinia sclerotiorum	30	Ss30	PRJNA418121	[3]
	Botrytis cinerea	12<br>18<br>24	Bc12<br>Bc18<br>Bc24	PRJNA315516<br>PRJNA593073	Utrecht University (2016)<br>Beijing normal University (2019)
	Control	12	Healthy12	PRJNA315516<br>PRJNA418121	[1]
		18	Healthy18		
		24	Healthy24		
		30	Healthy30		

HPI: hours post-inoculation

Table S2. Databases, bioinformatic tools and source code.

**Databases to extract RNA-Seq data and complementary data**		
**DB OR FILE NAME**	**DESCRIPTION**	**WEBSITE**
SRA-NCBI DB	Bulk RNASeq data	https://www.ncbi.nlm.nih.gov/sra
TAIR10 Genome Fasta File	TAIR10 genome release 2010	https://www.arabidopsis.org/download/index-auto.jsp?dir=/download_files/Sequences
Araport11 Genome Annotation	Genome Annotation Release 2016	https://www.arabidopsis.org/download/index-auto.jsp?dir=/download_files/Sequences
**Bioinformatic tools to estimate the RNA-Seq raw-counts**		
**TOOL NAME**	**DESCRIPTION**	**WEBSITE**
SRA-Toolkit	To download SRA accessions	Toolkithttps://hpc.nih.gov/apps/sratoolkit.html
FastQC	To perform quality analysis	https://www.bioinformatics.babraham.ac.uk
Trimmomatic	To preprocessing fq files	http://www.usadellab.org/cms/?page=trimmomat
STAR	To perform read alignments	https://github.com/alexdobin/STAR
HT-Seq-qa<br>HT-Seq-count	To test SAM files quality<br>To estimate raw-counts	https://htseq.readthedocs.io/en/master/
**4-Step methodology for RNA-Seq integration. Python (v3.8.10)**<br>https://github.com/cyntsc/RNA-Seq-raw-integration**. DOI 10.5281/zenodo.7076416**		
STEP 1: Raw-count integration	1_Step1_integrating_raw_counts (integrate and clean datasets)<br>Venn_diagram_genes_in_ceros.ipynb (compare TT vs CL repressed genes)	
STEP 2: TPM normalization	2_Step2_TPM_normalization.ipynb (normalize datasets)<br>*(prepare a gene length file)	
STEP 3: Data standardization	3_Step3_TPM_standardization.ipynb (cut off extreme and underrepresented values)	
STEP 4: Data log transformation and atypical identification	4_Step4_Log2_scale.ipynb (reduce scale representation and identify source of noise)	
Script to extract gene lengths for step 2.	Gene_length_extraction_from_GTF.ipynb (extract gene lengths for normalization)	
Script to extract gene modules for annotation tasks	6_modules_percentual_differentiation.ipynb	
**Network implementation and gene module identification. R (v4.1.0)**<br>https://github.com/cyntsc/RNA-Seq-raw-integration**. DOI 10.5281/zenodo.7076416**		
01_Healthy_SignedNtw_D.R<br>01_Infected_SignedNtw_E.R<br>02_Healthy_GS_MM_24hpi.R<br>02_Infected_GS_MM_24hpi.R	Network implementation for a signed-ntw for the healthy plants<br>Network implementation for a signed-ntw for the infected plants<br>Gene-Significance and Module-Membership for the healthy network<br>Gene-Significance and Module-Membership for the infected network	
**Gene Ontology annotation**		
Panther v17.0 (Released 20221013)	http://pantherdb.org/webservices/go/overrep.jsp	

Table S3. Significant gene modules identified in the healthy-control and fungal-infected samples.

Condition	Module Name	Cluster Size	R^2
Control samples gene networks	coral3 *	2024	+0.98
	blue3 *	79	+0.96
	navajowhite3 *	492	+0.79
	magenta2	204	-0.98
	darkolivegreen4	1792	-0.97
	antiquewhite	764	-0.88
Stress-treated gene networks	chocolate *	818	+0.98
	dodgerblue1 *	72	+0.96
	green3 *	472	+0.95
	chocolate2 *	573	+0.91
	tomato2	218	-0.94
	palevioletred1	368	-0.93
	green	236	-0.90
	deepskyblue	39	-0.80

* Gene modules with positive R^2 were selected to perform the Gene Ontology overrepresentation gene test.

Table S4. Gene Ontology overrepresentation test summary.

	Module name	#Genes	GO Category	Mapped genes	Classified	Unclassified
**Control samples gene networks**	Coral3	2024	MF	1991	726	1265
	navajowhite3	492	BP	489	168	321
	Blue3	79	BP	79	76	3
**Stress-treated gene networks**	Chocolate	818	MF	805	247	558
	Chocolate2	573	MF	566	180	386
	Green3	472	MF	456	118	338
	Dodgerblue1	72	None	70	NA	NA

MF: Molecular function, BP: Biological Process

Table S5. Genes functional groups related to multiple fungal infections identified by DAVID

**Group Description**	**Number of genes (including duplicates)**	**EASE Score* (>0.8)**
Cell wall / extracellular region	28	0.42835
Developmental protein	23	0.59716
Glycosyltransferase	34	**1.34897**
Methyltransferase	13	**0.87954**
Microtubule binding	11	0.77645
Protein kinase activity	85	0.44928
Protein transport	23	**1.11126**
Regulation of transcription	75	0.31824
Sterol metabolism	22	**1.35113**
WD40 repeat / G-protein	28	**1.55271**
Zinc finger	17	**1.01733**
mRNA binding	14	0.62084
LRR Receptors	15	0.52845

*Scores highlighted in bold show the groups selected in this study.

References

1. O'Connell, R.J.; Thon, M.R.; Hacquard, S.; Amyotte, S.G.; Kleemann, J.; Torres, M.F.; Damm, U.; Buiate, E.A.; Epstein, L.; Alkan, N.; et al. Lifestyle Transitions in Plant Pathogenic Colletotrichum Fungi Deciphered by Genome and Transcriptome Analyses. *Nat Genet* **2012**, *44*, 1060–1065, doi:10.1038/ng.2372.
2. Robin, G.P.; Kleemann, J.; Neumann, U.; Cabre, L.; Dallery, J.-F.; Lapalu, N.; O'Connell, R.J. Subcellular Localization Screening of Colletotrichum Higginsianum Effector Candidates Identifies Fungal Proteins Targeted to Plant Peroxisomes, Golgi Bodies, and Microtubules. *Front. Plant Sci.* **2018**, *9*, 562, doi:10.3389/fpls.2018.00562.
3. Badet, T.; Voisin, D.; Mbengue, M.; Barascud, M.; Sucher, J.; Sadon, P.; Balagué, C.; Roby, D.; Raffaele, S. Parallel Evolution of the POQR Prolyl Oligo Peptidase Gene Conferring Plant Quantitative Disease Resistance. *PLOS Genetics* **2017**, *13*, e1007143, doi:10.1371/journal.pgen.1007143.
